# Chemical profiling and anticancer activity of *Alnus incana* dichloromethane fraction on HeLa cells via cell cycle arrest and apoptosis

**DOI:** 10.1186/s12906-025-04920-z

**Published:** 2025-05-26

**Authors:** Walaa Hesham, Emad M. Elzayat, Mohamed Hosney, Fatma Abo-Elghiet

**Affiliations:** 1 Allergy and Immunology Lab, Vacsera, Giza Egypt; 2 Biotechnology Department, Faculty of Science, Cairo University, Giza, Egypt; 3 Zoology Department, Faculty of Science, Cairo University, Giza, Egypt; 4 Pharmacognosy and Medicinal Plants Department, Faculty of Pharmacy (Girls), Al- Azhar University, Cairo, Egypt

**Keywords:** *Alnus incana*, Cervical cancer, HeLa cells, Cytotoxicity, Cell cycle arrest, Apoptosis

## Abstract

**Background:**

Cervical cancer remains a global health challenge with persistently high incidence and mortality rates despite advancements in conventional treatments. The therapeutic potential of natural products has gained attention, particularly for their selective cytotoxicity and ability to modulate cancer pathways. *Alnus incana* (L.) Moench, a species-rich in bioactive compounds, shows potential as an anticancer agent; however, the cytotoxic effects of its leaves dichloromethane (DCM) extract remain underexplored. This study investigates the DCM fraction’s cytotoxicity on various cancer cell lines, with a primary focus on HeLa cells.

**Methods:**

The cytotoxic effects of the *A. incana* DCM fraction were evaluated in a dose-dependent manner using the MTT assay on several cancer cell lines, with particular emphasis on HeLa cells. Flow cytometry was used to assess cell cycle arrest and apoptosis, while RT-qPCR quantified changes in the expression of apoptotic markers (*Bax*,* Bcl-2*, and *p53*). Chemical composition analysis was conducted using gas chromatography-mass spectrometry/flame ionization detection (GC-MS/FID) to identify the major bioactive compounds within the fraction.

**Results:**

The DCM fraction exhibited dose-dependent cytotoxicity in HeLa cells, with an IC_50_ value of 135.6 µg/mL and a selectivity index (SI) of 2.72 relative to normal cells. Flow cytometry analysis revealed G0/G1 cell cycle arrest, significantly hindering progression through the S and G2/M phases. Moreover, there was a significant increase in both early and late apoptotic cell populations, correlating with the upregulation of pro-apoptotic genes (*Bax* and *p53*) and the downregulation of the anti-apoptotic gene *Bcl-2*. The chemical analysis identified 22 compounds in the unsaponifiable fraction, chiefly terpenoids such as phytol (65.74%). The saponifiable fraction presented a balanced composition of saturated (48.69%) and unsaturated (51.29%) fatty acids, with palmitic acid, linolenic acid, and linoleic acid as the predominant compounds.

**Conclusion:**

While the DCM fraction’s relatively high IC_50_ value may limit its utility as a standalone treatment, its ability to induce cell cycle arrest and apoptosis demonstrates its promise as a co-therapeutic agent with conventional anticancer drugs. Further research is essential to elucidate its precise mechanisms of action and to evaluate its efficacy in combination therapies, potentially advancing its role in cervical cancer treatment.

**Supplementary Information:**

The online version contains supplementary material available at 10.1186/s12906-025-04920-z.

## Introduction

Cancer, in its diverse forms, remains a significant global health burden, characterized by high incidence and mortality rates. Cervical cancer, a primary type of gynecological cancer predominantly caused by high-risk human papillomavirus (HPV) infection, is a striking example of this burden. Additional risk factors, including HIV (human deficiency virus), *Chlamydia trachomatis*, smoking, and hormonal influences (e.g. high parity and long-term oral contraceptive use), amplify its aggressiveness and contribute to its widespread impact on women’s health worldwide [1]. Despite advancements in treatment, which typically combines platinum-based chemotherapy (e.g. cisplatin) with adjuvant radiation [2, 3], cervical cancer remains the fourth most common cancer in women globally, with an estimated 660,000 new cases and 350,000 deaths in 2022 [1]. This ongoing challenge underscores the urgent need for adjunctive therapies that selectively target cancer cells while minimizing toxicity to healthy tissues and potentially improving patient quality of life [4, 5].

Natural products, known for their diverse bioactive compounds and favorable safety profiles, are emerging as promising candidates to fill this therapeutic gap. Many exhibit anticancer properties through modulation of key cancer pathways, including the AKT/mTOR pathway, apoptosis, and cell cycle regulation [6, 7]. *Alnus incana* (L.) Moench (*A. incana*), a member of the Betulaceae family, belongs to a genus with a rich history of traditional medicinal use for various ailments, including cancer [8]. This genus, comprising approximately 35 species, is known for producing a wide array of bioactive constituents, including triterpenoids, steroids, flavonoids, phenolics, tannins, diarylheptanoids, and other potentially beneficial compounds [8, 9]. While studies have evaluated cytotoxic activity in *A. incana* water and methanol extracts [10, 11], the cytotoxic potential of the dichloromethane (DCM) extract remains unexplored. Interestingly, Rashed et al. reported superior cytotoxic activity in the DCM extract of *Alnus rugosa*, a subspecies closely related to *A. incana* [8], suggesting that *A. incana* DCM extract may also possess similar potent anticancer properties with potential benefits for gynecological cancer treatment.

Inducing apoptosis and cell cycle arrest are well-established strategies in cancer treatment, particularly gynecological cancers, as they enable selective targeting of tumor cells while reducing harm to reproductive and other healthy tissues [12]. Apoptosis, a controlled process of cell death, is frequently disrupted in cancer cells, enabling them to evade elimination and continue proliferation. Therapeutic approaches that target apoptotic pathways, such as *BCL-2* inhibitors that block survival signals and *TRAIL* analogues that trigger death receptors, aim to reinstate the apoptotic response in resistant tumor cells. Additionally, drugs targeting key regulators of apoptosis, such as the *p53* and ISR signaling pathways, show promise in restoring normal cell death processes and limiting tumor growth [13]. Similarly, inducing cell cycle arrest can prevent cancer cells from dividing, effectively halting tumor progression. By disrupting the cell cycle at critical checkpoints, these therapeutic agents compel cancer cells to pause, leading to eventual cell death. This approach has shown effectiveness across a range of cancers, with several cell cycle-targeting drugs demonstrating clinical success [14]. Recent studies highlight the efficacy of various natural extracts in inducing these mechanisms, offering promising avenues for cancer therapeutic development [13]. Investigating natural products may unveil novel compounds that selectively disrupt these critical pathways, providing solutions to the persistent challenges of toxicity and drug resistance faced by current treatments, and potentially reducing chemotherapy-induced side effects, which could enhance overall patient well-being.

Therefore, this study aimed to investigate the cytotoxic effects of the DCM fraction of *A. incana* leaves on cervical cancer cells, particularly HeLa cells, a widely used model for gynecological cancers. The study examined the fraction’s impact on cell cycle progression, induction of apoptosis, and the expression of key genes (*Bax*,* p53*,* Bcl-2*) related to cell death pathways. Additionally, a comprehensive chemical profiling of the fraction was conducted to identify the bioactive constituents that may contribute to its cytotoxic properties. This research not only enhances understanding of potential adjunctive treatments for cervical cancer but also contributes to the broader field of complementary approaches in gynecological oncology, aiming to improve patient outcomes, quality of life, and overall well-being.

## Methods

### Plant material and extraction

The *A. incana* DCM fraction used in this study was obtained from our previous work [9]. In brief, *A. incana* (L.) Moench leaves were collected with permission from Al Zoharia Research Garden, Cairo, Egypt, following institutional, national, and international guidelines. Dr. Mamdouh Shokry, a botanist at Al Zoharia Research Garden, validated and authenticated the plant material. A voucher specimen was deposited at the herbarium of the Pharmacognosy Lab, Faculty of Pharmacy (Girls), Al-Azhar University, under the number AR-2016. The leaves were extracted with 100% methanol, followed by liquid-liquid partitioning using solvents of increasing polarity. The DCM fraction, representing the lipoidal portion, was selected for further investigation in this study.

### Cell lines and culture condition

Human cancer cell lines, HepG2 (liver), MCF-7 (breast), HCT116 (colon), and HeLa (cervical), along with the non-cancerous HSF (human skin fibroblasts) cell line were obtained from American Type Cell Culture Collection (ATCC) and maintained as adherent monolayers at VACSERA cell culture library (Giza, Egypt). All cells were cultured in RPMI-1640 medium (Lonza GmbH, Cologne, Germany) supplemented with 10% heat-inactivated fetal bovine serum (FBS; BIOWEST, Bradenton, FL, USA), 100 U/mL penicillin (Company), and 2 mg/mL streptomycin (Company). Cells were incubated at 37 °C in a humidified atmosphere with 5% CO_2_, and regular subculturing was performed under aseptic conditions to maintain exponential cell growth.

### MTT assay

Cell viability was assessed using the MTT assay as described by [15]. Briefly, this colorimetric method measures the reduction of a yellow tetrazolium salt (MTT) to a purple formazan product by metabolically active cells. Briefly, cells were seeded at a density of 50,000 cells/well in 100 µL of the media in a 96-well plate and incubated for 24 h. Afterward, serial concentrations of the *A. incana* DCM fraction (400–3.1 µg/mL) and doxorubicin (20–2.5 µg/mL, positive control) were added, followed by a 48-hour incubation. Four wells were used for each condition, and the experiment was repeated independently three times to ensure reproducibility. Doxorubicin was selected as the positive control due to its well-established efficacy and its use as a benchmark in cancer research. Its potent cytotoxic effects make it a standard reference for evaluating the antiproliferative activity of novel compounds. Next, 10 µL of MTT solution was added to each well, and the plate was incubated for 4 h at 37 °C. The formed formazan crystals were dissolved in 100 µL DMSO with agitation, and absorbance was measured at 570 nm using an ELX800 UV universal microplate reader. Cell viability was calculated as a percentage of the control using the formula:

(Mean absorbance of treated sample / Mean absorbance of control) × 100.

Dose-response curves were generated, and IC_50_ (half-maximal inhibitory concentration) was determined using Prism software. The selectivity index (SI) was calculated as the ratio of the IC_50_ value for normal cells to the IC_50_ value for cancer cells, with SI > 1 indicating selectivity towards cancer cells. Higher SI values suggest greater therapeutic selectivity and potential efficacy [16].

### Cell cycle analysis

To assess the impact of *A. incana* DCM fraction on cell cycle progression, propidium iodide (PI) staining followed by flow cytometry was employed as outlined previously [17]. PI (Company) is a fluorescent dye that binds to DNA allowing differentiation of cell cycle phases (G0/G1, S, G2/M) based on their DNA content. Briefly, HeLa cells (2 × 10^5^ cells/mL) were cultured overnight and then treated with the DCM fraction at its IC_50_ concentration (135.6 µg/mL) for 24 h. After treatment, cells were fixed, stained with a PI/RNase solution, and analyzed using a FACSCalibur Scan flow cytometer (BD Biosciences) to determine cell cycle distribution.

### Apoptosis analysis using Annexin V-FITC/PI assay

To determine the mode of cell death induced by *A. incana* DCM fraction, the Annexin V-FITC apoptosis detection kit (BioVision Inc., CA, USA, K101-25) was used following the manufacturer’s protocol. This assay detects phosphatidylserine translocation, a hallmark of early apoptosis, by Annexin V-FITC staining. PI, a viability dye excluded by healthy and early apoptotic cells with intact membranes, stains the DNA of late apoptotic and necrotic cells with compromised membranes, allowing for their identification. Briefly, HeLa cells (2 × 10^5^ cells/mL) were cultured for 24 h and treated with the extract at its IC_50_ concentration (135.6 µg/mL) for an additional 48 h. Following treatment, cells were stained with Annexin V-FITC and PI. Apoptosis was quantified by flow cytometry using a BD FACSCalibur Scan system (BD Biosciences).

For accurate quantification of apoptotic populations, a specific gating strategy was applied. Cells were initially gated based on forward scatter (FSC) versus side scatter (SSC) to exclude debris, followed by gating on FSC-Height (FSC-H) versus FSC-Area (FSC-A) to exclude doublets. Apoptotic populations were classified by gating on Annexin V-FITC versus PI as follows: live cells (Q4) were Annexin V-/PI-, early apoptotic cells (Q3) were Annexin V+/PI-, late apoptotic/necrotic cells (Q2) were Annexin V+/PI+, and dead/necrotic cells (Q1) were Annexin V-/PI+. The percentage of cells in each quadrant was calculated to assess the extent of apoptosis and necrosis.

### RNA extraction and Real-time qPCR

Total RNA was isolated from both treated and untreated HeLa cells using the RNeasy Mini Kit (Qiagen, 74104) following the manufacturer’s protocol. To evaluate the effects of *A. incana* DCM extract (135.6 µg/mL, IC_50_) on gene expression, complementary DNA (cDNA) was synthesized from 1 µg of RNA using the iScript™ One-Step RT-PCR Kit (Bio-Rad, Hercules, CA). Primers for the target genes (Table 1) were verified with NCBI Primer-Blast. Relative expression levels were normalized to GAPDH as the housekeeping gene and calculated using the 2^−ΔΔCt^ method. The PCR conditions included an initial denaturation at 50 °C for 10 min, and 95 °C for 5 min, followed by 30–45 cycles of 95 °C for 10 s and 55–60 °C for 30 s. A melting curve was generated by gradually increasing the temperature from 60 °C to 95 °C after the final cycle.

**Table 1:** Primer pair sequences used for qRT-PCR analysis

Genes	Primers
*Bcl-2*	F 5’- ATCGCCCTGTGGATGACTGAGT − 3’ R 5’- GCCAGGAGAAATCAAACAGAGGC-3’
*P53*	F 5’- CCTCAGCATCTTATCCGAGTGG − 3’ R 5’- TGGATGGTGGTACAGTCAGAGC − 3’
*Bax*	F 5’-TCAGGATGCGTCCACCAAGAAG − 3’ R 5’-TGTGTCCACGGCGGCAATCATC − 3’
*GAPDH*	F 5’- GTCTCCTCTGACTTCAACAGCG − 3’ R 5’- ACCACCCTGTTGCTGTAGCCAA-3’

### Chemical profile of *A. incana* DCM fraction

The *A. incana* DCM fraction was subjected to comprehensive chemical analysis using GC-MS/FID detection. GC-MS was employed to identify unsaponifiable compounds, while GC-FID was utilized for fatty acids characterization.

For GC-MS analysis, the DCM fraction underwent saponification with ethanolic potassium hydroxide, followed by extraction of the unsaponifiable matter with toluene. The extract was injected into a GC-MS system (Agilent 7890B) equipped with a DB-5MS column (30 m x 0.25 mm ID, 0.25 μm film thickness). Helium served as the carrier gas at a flow rate of 3.0 mL/min in splitless mode, with an injection volume of 1 µL. The temperature program was as follows: 40 °C for 1 min, increased by 10 °C/min to 200 °C (held for 1 min), then increased by 20 °C/min to 220 °C (held for 1 min), and finally increased by 30 °C/min to 320 °C (held for 10 min). The injector and detector temperatures were set at 250 °C and 320 °C, respectively. Mass spectra were obtained using electron ionization (70 eV) in the *m/z* range of 50–600, with compound identification performed by comparing spectra to the Wiley and NIST Mass Spectral Libraries.

For fatty acid analysis, the saponifiable matter was derivatized into fatty acid methyl esters (FAMEs) using a 2 M potassium hydroxide solution in methanol. The FAMEs were injected into a GC system (Agilent 7890B) equipped with a Zebron ZB-FAME column (60 m x 0.25 mm ID, 0.25 μm film thickness). Hydrogen was used as the carrier gas at a flow rate of 1.8 mL/min (split 1:50), with an injection volume of 1 µL. The temperature program began at 100 °C for 3 min and increased by 2.5 °C/min to 240 °C (held for 10 min). The injector and flame ionization detector (FID) were set at 250 °C and 285 °C, respectively. Fatty acids were identified by comparing the retention times of FAME peaks with those of known standards.

### Statistical analysis

Statistical analysis was performed using SPSS version 24.0. Differences between the means of two independent groups were analyzed using an independent t-test, with *p* < 0.05 (*) considered statistically significant, indicating meaningful differences in the data.

## Results

### Cytotoxicity and antiproliferative activity of *A. incana* DCM fraction

**Fig. 1:**
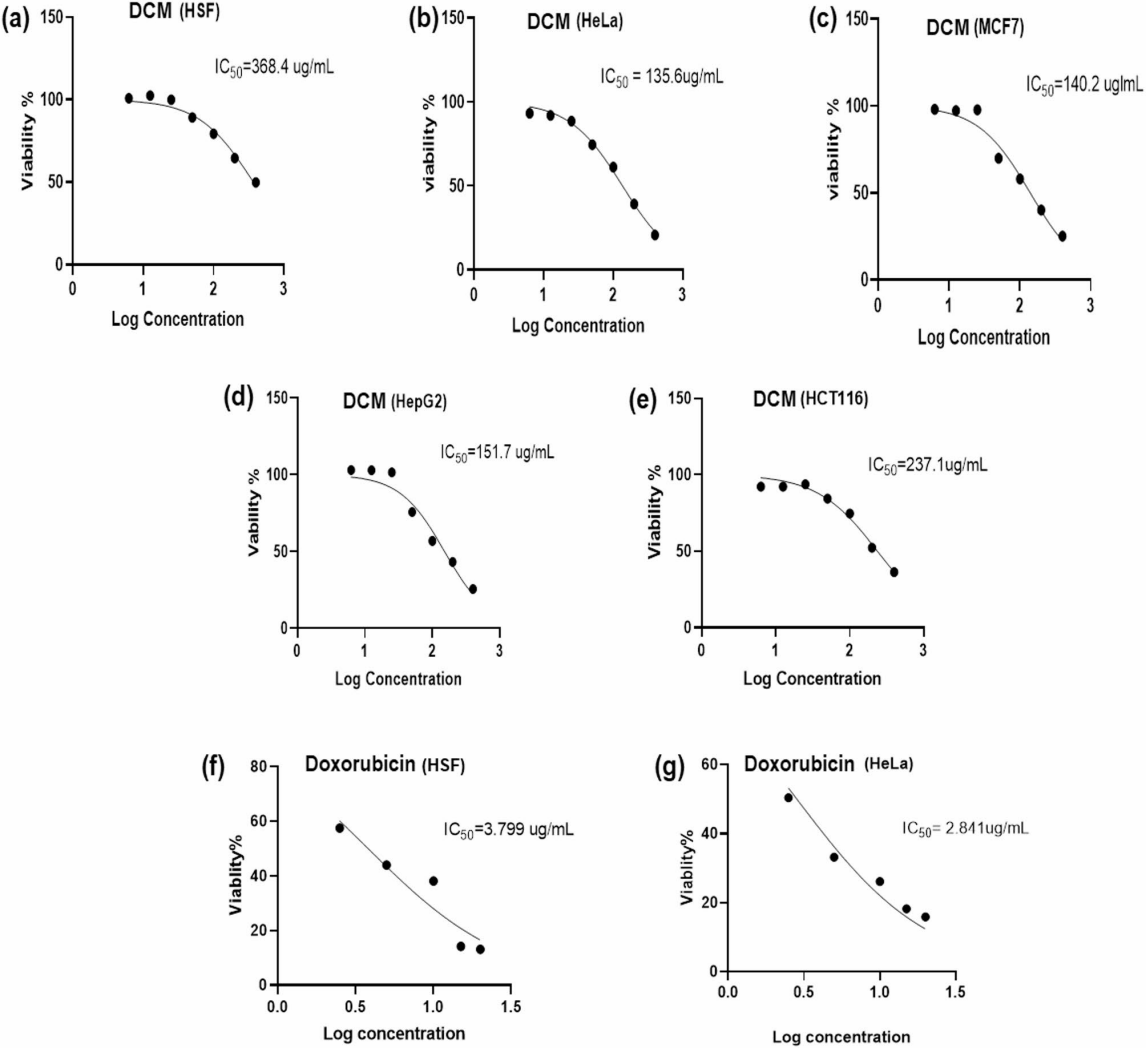
Cytotoxic effects and IC_50_ values of the *A. incana* DCM fraction and doxorubicin on various cell lines over 48 h, as determined by the MTT assay. Panels (**a**–**e**) show the cytotoxic effects of the *A. incana* DCM fraction on (**a**) normal human skin fibroblasts (HSF), (**b**) cervical cancer cells (HeLa), (**c**) breast cancer cells (MCF-7), (**d**) liver cancer cells (HepG2), and (**e**) colon cancer cells (HCT-116). Panels (**f**) and (**g**) illustrate the cytotoxic effects and IC_50_ values of doxorubicin on (**f**) HSF and (**g**) HeLa cells after 48 h

To evaluate the cytotoxic potential and selectivity of the *A. incana* DCM fraction, MTT assay was performed on four cancer cell lines (HepG2, MCF-7, HCT116, and HeLa) along with a normal cell line (HSF). The DCM fraction exhibited a dose-dependent inhibition of cell proliferation across all tested cancer cell lines, with IC_50_ values ranging from 135.6 to 237.1 µg/mL (Fig. 1; Table 2). To assess the selectivity, the Selectivity Index (SI), calculated as the IC_50_ ratio in normal cells to cancer cells, was determined (Table 2). Based on the IC_50_ and SI values, the HeLa cell line was selected for further investigation due to its favorable response. Furthermore, the IC_50_ and SI of doxorubicin against HeLa cells were also determined as a positive control (Fig. 1; Table 2).

**Table 2:** IC_50_ values and selectivity index (SI) of *A. incana* DCM fraction and doxorubicin against various cell lines

Cell Line	A. incana DCM fraction		Doxorubicin	
	IC_50_ (µg/mL) ± SD	SI	IC_50_ (µg/mL) ± SD	SI
HSF (Normal)	368.4 ± 16.57	--	3.8 ± 0.076	--
HeLa	135.6 ± 6.78	2.72	2.8 ± 0.196	1.33
MCF-7	140.2 ± 9.81	2.63	--	--
HepG2	151.7 ± 9.10	2.43	--	--
HCT116	237.1 ± 19.96	1.55	--	--

### Effect of *A. incana* DCM fraction on cell cycle analysis

**Fig. 2:**
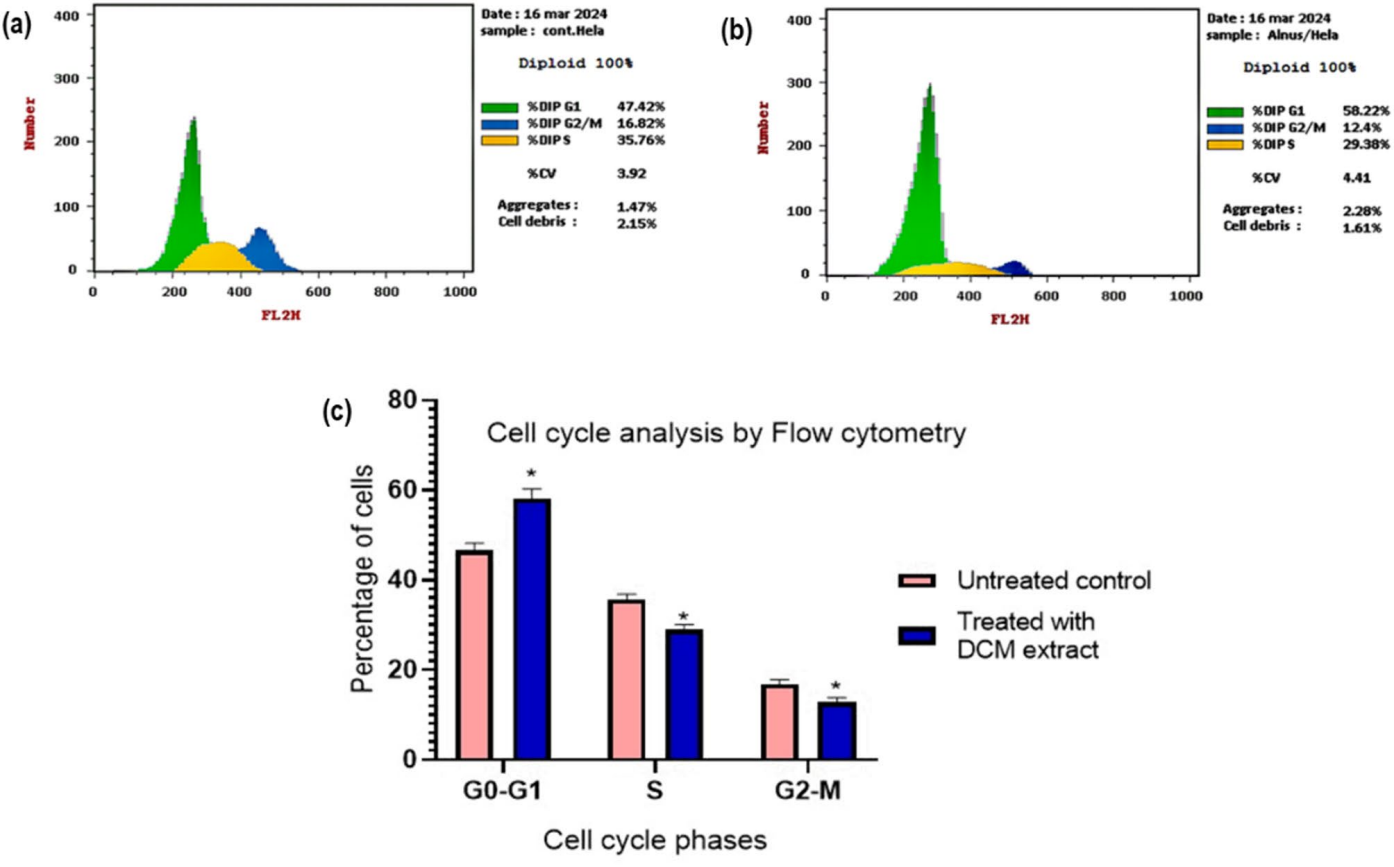
Flow cytometric analysis of cell cycle distribution in HeLa cells treated with the *A. incana* DCM fraction. HeLa cells were treated with the IC_50_ concentration (135.6 µg/mL) of the *A. incana* DCM fraction for 48 h, and cell cycle phase distribution was analyzed via flow cytometry. Panels: (**a**) Control (untreated) HeLa cells, (**b**) HeLa cells treated with the DCM fraction, and (**c**) A graphical comparison of cell percentages across each cell cycle phase between treated and untreated groups

To investigate the effect of the *A. incana* DCM fraction on cell cycle progression, HeLa cells were treated with the IC_50_ concentration (135.6 µg/mL) of the fraction for 48 h. Cell cycle distribution was assessed by flow cytometry using Propidium Iodide (PI) staining, which binds to DNA and allows differentiation of cell cycle phases (G0/G1, S, G2/M) based on DNA content. As illustrated in Fig. 2, a significant increase (*p* < 0.05) in the G0/G1 phase population (58.22%) compared to the control (47.42%) was observed, indicating cell cycle arrest at the G0/G1 checkpoint. Concomitantly, significant decreases were observed in both the S phase (29.38%) and G2/M phase (12.4%) populations relative to untreated cells (35.76% and 16.82%, respectively). These results suggest that the DCM fraction of *A. incana* exerts cytostatic effects on HeLa cells by inducing cell cycle arrest.

### Effect of *A. incana* DCM fraction on apoptosis and necrosis in HeLa cells

**Fig. 3:**
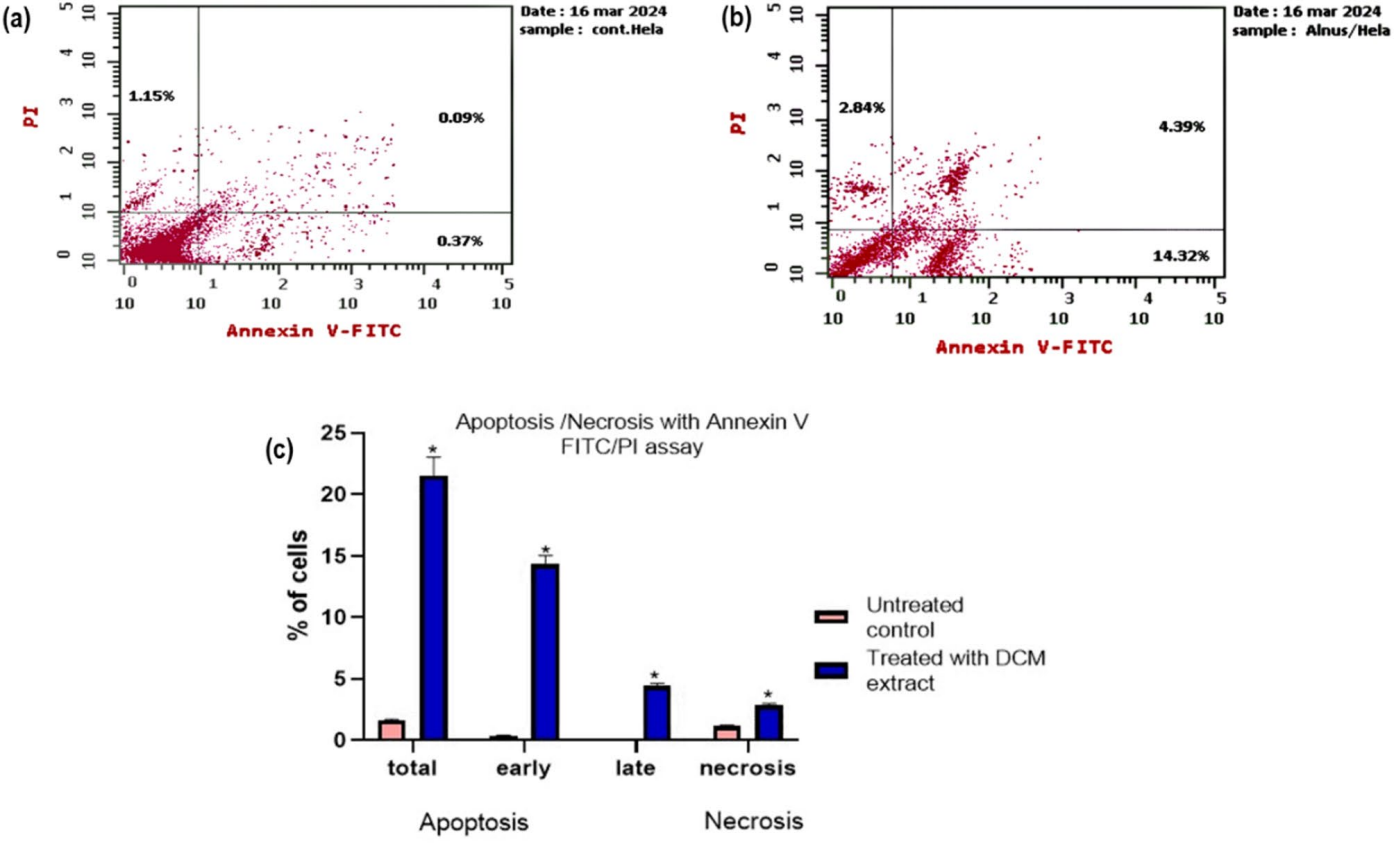
Flow cytometric analysis of apoptosis and necrosis in HeLa cells treated with the *A. incana* DCM fraction. Detection of apoptotic and necrotic cell populations was conducted using Annexin V-FITC and PI dual staining after treating HeLa cells with the IC_50_ concentration (135.6 µg/mL) of the *A. incana* DCM fraction for 48 h. Quadrant distribution: upper left (necrotic cells), lower left (viable cells), lower right (early apoptotic cells), and upper right (late apoptotic cells). Panels: (**a**) Control (untreated) HeLa cells, (**b**) DCM-treated HeLa cells, and (**c**) Graphical representation comparing cell percentages in each quadrant between treated and untreated cells

HeLa cells treated with the IC₅₀ concentration (135.6 µg/mL) of the *A. incana* DCM fraction for 48 h were assessed for apoptosis and necrosis using Annexin V-FITC and PI staining analyzed by flow cytometry. As illustrated in Fig. 3, treatment with the *A. incana* DCM fraction resulted in a statistically significant increase (*p* < 0.05) in early and late apoptotic, as well as necrotic cells, compared to the untreated control group. Specifically, the percentage of early apoptotic cells increased from 0.37 to 14.32%, and late apoptotic cells from 0.09 to 4.39%. Necrosis was also notably induced, with the proportion of necrotic cells rising from 1.15 to 2.84%.

### Gene expression

**Fig. 4:**
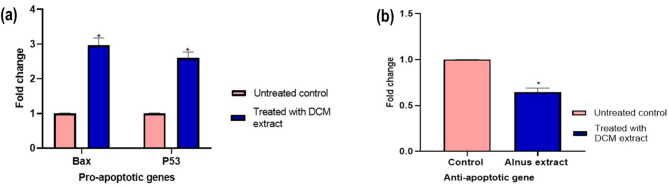
Gene expression of (**a**) Pro-apoptotic genes *p53* and *Bax*, and (**b**) Anti-apoptotic gene *Bcl-2* in HeLa cells treated and untreated with the *A. incana* DCM fraction

To investigate the molecular mechanisms underlying the apoptotic effects of the *A. incana* DCM fraction, reverse transcription-quantitative polymerase chain reaction (RT-qPCR) was employed to analyze the expression of key apoptosis-related genes in HeLa cells. Treatment with the IC_50_ concentration of the DCM extract significantly upregulated the expression of the pro-apoptotic genes *p53* and *Bax*, with fold change values of 2.60 and 2.96, respectively. In contrast, the expression of the anti-apoptotic gene *Bcl-2* was significantly downregulated, with a fold change value of 0.6467 (Fig. 4).

### Chemical profile of *A. incana* DCM fraction

**Fig. 5:**
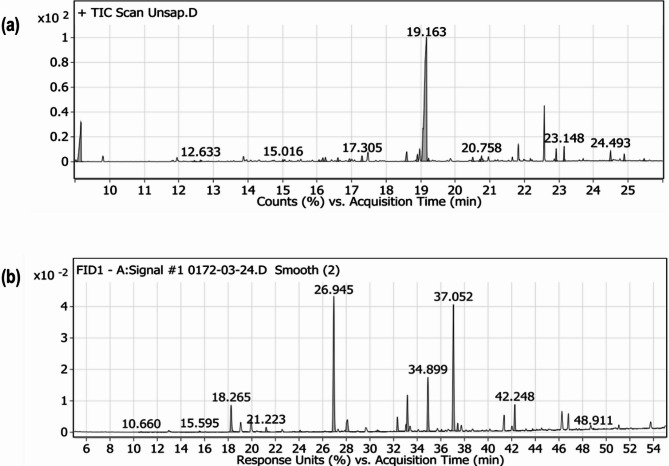
Chromatographic analysis of *Alnus incana* leaves’ DCM fraction: (**a**) GC-MS chromatogram for unsaponifiable matter, and (**b**) GC-FID chromatogram for saponifiable matter (fatty acids)

The chemical analysis of the *Alnus incana* leaves’ DCM fraction, conducted using GC-MS for unsaponifiable matter and GC-FID for saponifiable matter, revealed a diverse composition of compounds (Fig. 5). The unsaponifiable fraction was predominantly composed of terpenoids (75.07%), with phytol—a diterpene chlorophyll compound known for its potential therapeutic properties—as the most abundant component (65.74%). Other significant terpenoids included isophytol, squalene, lupeol, and lup-20(29)-en-3-one. Additionally, the fraction contained hydrocarbons, alcohols, ketones, esters, and sterols like γ-sitosterol (Table 3). The saponifiable fraction primarily consisted of fatty acids, nearly equally divided between saturated (48.69%) and unsaturated (51.29%) types. Among the saturated fatty acids, palmitic acid (31.27%) was the most abundant, while linolenic acid (26.98%) and linoleic acid (11.21%) were the major unsaturated fatty acids (Table 4). These findings underscore the substantial presence and potential pharmacological relevance of both terpenoids and fatty acids in the DCM fraction of *A. incana* leaves.

**Table 3:** Chemical profile of the unsaponifiable matter in the DCM fraction of *A. incana* leaves

Peak	RT	Name	Formula	% Area
Terpenoids and related compounds:				
1.	12.457	(-)-Aristolene	C_15_H_24_	0.33
2.	16.605	3,7,11,15-Tetramethylhexadec-2-en-1-yl acetate (Phytyl acetate)	C_22_H_42_O_2_	0.78
3.	16.942	1,6,10,14-Hexadecatetraen-3-ol, 3,7,11,15-tetramethyl-,(E, E)-	C_20_H_34_O	0.76
4.	17.305	Isophytol	C_20_H_40_O	1.21
5.	18.594	Phytyl,2-methylbutanoate	C_25_H_48_O_2_	2.87
6.	**19.163**	**Phytol**	**C**_**20**_ **H**_**40**_ **O**	**65.74**
7.	22.916	Squalene	C_30_H_50_	1.52
8.	24.887	Lupeol	C_30_H_50_O	1.32
9.	25.462	Lup-20(29)-en-3-one	C_30_H_48_O	0.54
Sterols:				
10.	24.493	γ-Sitosterol	C_29_H_50_O	1.92
Hydrocarbons:				
11.	15.016	Benzene, (1-pentylheptyl)-	C_18_H_30_	0.39
12.	16.060	Benzene, (1-pentyloctyl)-	C_19_H_32_	0.33
13.	23.148	Hentriacontane	C_31_H_64_	1.76
Alcohols and ketones:				
14.	9.023	3-Phenylpropan-1-ol	C_9_H_12_O	0.51
15.	16.167	1-Ethynylcyclododecanol	C_14_H_24_O	0.94
16.	9.167	2-Butanone,4-phenyl	C_10_H_12_O	13.56
17.	16.242	2-Pentadecanone, 6,10,14-trimethyl	C_18_H_36_O	0.91
18.	20.502	3-Pentanone,1-cyclohexyl-5-phenyl	C_17_H_24_O	0.91
Esters:				
19.	18.907	Methyl 10-trans,12-cis-octadecadienoate	C_19_H_34_O_2_	1.55
20.	20.708	hexadecyl acrylate	C_19_H_36_O_2_	0.39
21.	20.758	2-Butyl-6-phenethyltetrahydro-4 H-pyran-4-one	C_17_H_24_O_2_	1.42
Phenolic compounds:				
22.	12.633	2,4-Di-tert-butylphenol	C_14_H_22_O	0.33

**Table 4:** Chemical profile of the saponifiable matter in the DCM fraction of *Alnus incana* leaves

Peak	RT	Name	Area %
Saturated fatty acids:			
1.	10.66	Capric acid	0.08
2.	15.595	Lauric acid	0.26
3.	18.265	Tridecanoic acid	6.34
4.	21.223	Myristic acid	1.04
5.	24.087	Pentadecanoic acid	0.37
6.	**26.945**	**Palmitic acid**	**31.27**
7.	32.322	Stearic acid	2.89
8.	37.412	Arachidic acid	1.42
9.	42.248	Behenic acid	5.02
Unsaturated fatty acids:			
10.	28.092	Palmitoleic acid	4.02
11.	30.647	cis-10-Heptadecenoic acid	0.54
12.	33.16	Oleic acid	7.61
13.	34.899	Linoleic acid	11.21
14.	36.047	gamma-Linolenic acid	0.26
15.	**37.052**	**Linolenic acid**	**26.98**
16.	39.713	*cis*-11,14-Eicosadienoic acid	0.04
17.	40.637	Homo-γ-linolenic acid	0.11
18.	41.66	Arachidonic acid	0.06
19.	43.747	EPA	0.27
20.	48.911	DHA	0.19

## Discussion

This study investigated the cytotoxic potential of the DCM fraction from the leaves of the medicinal plant *A. incana*. Given the documented cytotoxic activity of the DCM extract from the related subspecies *A. rugosa* [8], we hypothesized that *A. incana* might exhibit similar effects. Although previous studies confirmed the cytotoxic properties of its methanol extract [10], the effects of the DCM fraction remain unexamined. To address this gap, the fraction’s cytotoxicity was evaluated against four human cancer cell lines: HepG2, HCT116, HeLa, and MCF-7, representing diverse cancer types. The *A. incana* DCM fraction demonstrated IC_50_ values ranging from 135.6 to 273.1 µg/mL, with HeLa cells (a cervical cancer model) showing the highest sensitivity (IC_50_ = 135.6 µg/mL). Significantly, the DCM fraction exhibited a selectivity index (SI) of 2.72 against HeLa cells, indicating preferential cytotoxicity toward cancer cells over normal cells and highlighting its therapeutic potential.

Although the IC_50_ values for *A. incana* were higher than those reported for the DCM extract of the related subspecies *A. rugosa* [8], this difference may be attributed to variations in the concentration or composition of active compounds in each extract. Despite this, both species showed their most potent cytotoxic effects against HeLa cells, suggesting a potential shared mechanism targeting cervical cancer cells specifically. Additionally, prior studies have reported that the methanol extract of *A. incana* leaves has a pronounced effect on HeLa cells (IC_50_ of 68.5 µg/mL) [10]. The selective cytotoxicity observed in both *A. incana* and *A. rugosa* DCM extracts, alongside the methanol extract’s activity, indicates the presence of bioactive compounds within these species that may preferentially disrupt HeLa cell function. This selective activity underscores the potential of *Alnus* species as promising candidates for developing targeted therapies against gynecological cancers, particularly cervical cancer. Given the substantial cytotoxic response observed, further investigation into the active compounds and mechanisms of action of the DCM fraction of *A. incana* leaves is warranted.

To explore the mechanisms underlying this cytotoxicity, we investigated the effects of *A. incana* DCM fraction on cell cycle progression and apoptosis in HeLa cells. The results revealed a significant accumulation of cells in the G0/G1 phase, indicating effective cell cycle arrest. This is a crucial finding science cell cycle arrest at key checkpoints is a well-established therapeutic strategy for inhibiting cancer cell proliferation. The mammalian cell cycle comprises four distinct phases: G1, S, G2, and M, where RNA and protein synthesis occur in G1 and G2, DNA replication in S, and chromosome segregation in M [18]. By halting cells in the G0/G1 phase, the *A. incana* DCM fraction appears to block progression to DNA synthesis, which could lead to senescence or cell death. Several plant-derived anticancer agents, such as taxanes and vinca alkaloids, function by disrupting key cell cycle regulators [19].

In addition to inducing cell cycle arrest, the DCM fraction significantly promoted apoptosis, a fundamental mechanism in cancer treatment. Apoptosis, or programmed cell death, is triggered by many natural cytotoxic agents, both clinically approved and experimental, and is essential for eliminating cancer cells [20]. Flow cytometric analysis using Annexin V and propidium iodide staining revealed a marked increase in apoptotic cell death (40.67-fold increase, 18.71% vs. 0.46% in the control) induced by the *A. incana* DCM fraction. Early apoptosis rose to 14.32% from 0.37%, and late apoptosis increased to 4.39% from 0.09%. Necrosis also increased by 2.5-fold (2.84% vs. 1.15% in the control), indicating a broad cytotoxic effect on HeLa cells. These findings reinforce the potential of *A. incana* DCM fraction as an effective anticancer agent by simultaneously inducing cell cycle arrest and apoptosis.

To elucidate the molecular mechanisms behind these effects, we evaluated the expression of apoptotic regulators, including *p53*,* Bcl-2*, and *Bax*, through RT-PCR. The intrinsic apoptotic pathway is regulated by the balance between pro-apoptotic *Bax* and anti-apoptotic *Bcl-2*, where *Bcl-2* stabilizes the mitochondrial membrane and prevents cytochrome c release, whereas *Bax* promotes apoptosis by facilitating cytochrome c release [21]. *p53*, a critical tumor suppressor, enhances *Bax* expression, thus its stimulation tipping the balance towards apoptosis [22]. In our study, the *A. incana* DCM fraction significantly upregulated *Bax* and *p53* while downregulating *Bcl-2*, resulting in an elevated *Bax/Bcl-2* ratio, a key indicator of apoptosis activation. Previous research reported that elevated *p53* expression and a higher *Bax/Bcl-2* ratio correlate with increased tumor sensitivity to anticancer therapies [23], further supporting the potential of the *A. incana* DCM fraction as a therapeutic agent.

Given these findings, it is essential to contextualize the anticancer activity of the *A. incana* DCM fraction by comparing it to established cervical cancer treatments, such as cisplatin and paclitaxel. Cisplatin exerts its cytotoxic effects primarily through DNA cross-linking, causing extensive DNA damage that leads predominantly to G2/M cell cycle arrest and subsequent apoptosis induction mainly via *p53*-dependent pathways [24, 25]. Despite its efficacy, cisplatin is often limited by severe adverse effects, including nephrotoxicity, neurotoxicity, and drug resistance [25]. Paclitaxel, another cornerstone treatment, acts by stabilizing microtubules, causing prolonged G2/M mitotic arrest and promoting apoptosis through a *p53*-independent pathway involving cyclin B1/CDC2 activation and *Bcl-2* phosphorylation [24, 26]. However, paclitaxel similarly has clinical limitations, including dose-dependent toxicities such as peripheral neuropathy and myelosuppression [27]. In contrast, our findings demonstrate that the *A. incana* DCM fraction distinctly arrests cells at the G0/G1 phase and promotes apoptosis via a *p53*-dependent pathway. These unique molecular characteristics suggest a complementary role alongside traditional chemotherapeutics, potentially reducing toxicities and overcoming drug resistance, despite its relatively high IC_50_.

The chemical profile of the *A. incana* DCM fraction, analyzed via GC-MS/FID detection, revealed a high content of phytol (65.74%) in unsaponifiable matter, a compound known for its broad-spectrum biological activities, particularly in cancer therapy. Phytol, a diterpenoid derived from chlorophyll, has been widely recognized for its anti-inflammatory and anticancer properties, including the modulation of cell cycle and apoptosis pathways [28]. Previous studies have demonstrated its ability to induce apoptosis across various cancer cell lines, including lung, colon, and breast cancers, primarily through the disruption of mitochondrial membrane potential and subsequent activation of the intrinsic apoptotic pathway [28, 29]. Our findings, which show significant G0/G1 cell cycle arrest and apoptosis in HeLa cells, align with these established mechanisms of action, further validating phytol’s role as a potent anticancer agent. In addition to phytol, the saponifiable matter of the DCM fraction contained palmitic acid (31.27%) and linolenic acid (26.98%) as the major components. Both fatty acids are well-documented for their apoptosis-inducing capabilities. Palmitic acid, for instance, has been reported to upregulate pro-apoptotic proteins such as *Bax* and *p53*, while downregulating anti-apoptotic *Bcl-2*, leading to enhanced apoptosis via caspase activation in colorectal and breast cancer models [30]. Similarly, linolenic acid exerts anticancer effects by modulating the *Bcl-2* family proteins, increasing pro-apoptotic *Bax* expression, and inducing endoplasmic reticulum (ER) stress, which contributes to cancer cell death. Linolenic acid has also been shown to inhibit the PI3K/Akt signaling pathway and suppress fatty acid synthase (*FASN*), an enzyme overexpressed in many cancers [31]. Our study’s findings of increased *Bax* expression and a heightened *Bax/Bcl-2* ratio in HeLa cells are consistent with these mechanisms, suggesting that palmitic and linolenic acids likely contribute to the cytotoxic effects observed.

Despite these promising results, the study possesses limitations typical of in vitro investigations. Notably, the biological complexity of tumor microenvironments, such as gene expression variability, signaling heterogeneity, immune interactions, and stromal influences, cannot be adequately mimicked using isolated cell lines like HeLa. Additionally, the relatively high IC_50_ necessitates exploration of advanced drug delivery systems or combination therapies. Future research should incorporate additional cancer cell lines, normal cell toxicity assessments, patient-derived xenografts (PDX), and innovative delivery strategies, such as nanoparticle encapsulation, to enhance efficacy, reduce toxicity, and facilitate clinical translation. These steps are crucial for validating the preclinical promise and advancing the clinical applicability of the *Alnus incana* DCM fraction.

## Conclusion

This study demonstrates that the *A. incana* DCM fraction, rich in bioactive compounds such as phytol, palmitic acid, and linolenic acid, exerts notable cytotoxic effects on various cancer cell lines, particularly HeLa cells. These effects are mediated through inducing G0/G1 cell cycle arrest and apoptosis via modulation of *Bax*,* Bcl-2*, and *p53* pathways. Although the relatively high IC_50_ value (135.6 µg/mL) limits its potential as a standalone therapy, the fraction’s selective cytotoxicity toward cancer cells suggests a potentially favorable safety profile. Its unique ability to activate apoptotic and cell-cycle arrest pathways supports its promise as an adjunctive therapy, particularly in combination with conventional chemotherapeutic agents, which may improve treatment outcomes by reducing associated toxicities or overcoming resistance. Further in-depth mechanistic studies, along with rigorous in vivo evaluations using clinically relevant models such as patient-derived xenografts, are essential to validate these promising findings and fully realize the clinical potential of this natural fraction.

## Electronic supplementary material

Below is the link to the electronic supplementary material.

Supplementary Material 1

Supplementary Material 2

Supplementary Material 3

## Data Availability

Data is provided within the manuscript.

## Abbreviations

ATCC: American Type Culture Collection
BCL: B-cell Lymphoma (e.g., *BCL-2*, a family of apoptosis regulators)
DCM: Dichloromethane
DMSO: Dimethyl Sulfoxide
DNA: Deoxyribonucleic Acid
ER: Endoplasmic Reticulum
FAME: Fatty Acid Methyl Esters
FASN: Fatty Acid Synthase
FBS: Fetal Bovine Serum
FID: Flame Ionization Detector
FITC: Fluorescein Isothiocyanate
GAPDH: Glyceraldehyde-3-phosphate Dehydrogenase (housekeeping gene)
GC: Gas Chromatography
HIV: Human Immunodeficiency Virus
HPV: Human Papillomavirus
MCF: Michigan Cancer Foundation (MCF-7 breast cancer cell line)
MS: Mass Spectrometry
MTT: 3-(4,5-dimethylthiazol-2-yl)-2,5-diphenyltetrazolium bromide (assay reagent)
NCBI: National Center for Biotechnology Information
NIST: National Institute of Standards and Technology
PCR: Polymerase Chain Reaction
PI: Propidium Iodide
RNA: Ribonucleic Acid
RT: Reverse Transcription (in qRT-PCR)
SI: Selectivity Index
SPSS: Statistical Package for the Social Sciences
TRAIL: Tumor Necrosis Factor-Related Apoptosis-Inducing Ligand
UV: Ultraviolet (UV absorbance measurement)

## References

[1] Bray F, Laversanne M, Sung H, Ferlay J, Siegel RL, Soerjomataram I, et al. Global cancer statistics 2022: GLOBOCAN estimates of incidence and mortality worldwide for 36 cancers in 185 countries. CA: A Cancer Journal for Clinicians; 2024. 10.3322/caac.21834 38572751

[2] Burmeister CA, Khan SF, Schäfer G, Mbatani N, Adams T, Moodley J, et al. Cervical cancer therapies: current challenges and future perspectives. Tumour Virus Res. 2022;13:200238. 35460940 10.1016/j.tvr.2022.200238 PMC9062473

[3] Dasari S, Tchounwou PB. Cisplatin in cancer therapy: molecular mechanisms of action. Eur J Pharmacol. 2014;740:364–78. 25058905 10.1016/j.ejphar.2014.07.025 PMC4146684

[4] Romero SA, Pavan ICB, Morelli AP, Mancini MCS, da Silva LGS, Fagundes I, et al. Anticancer effects of root and beet leaf extracts (Beta vulgaris L.) in cervical cancer cells (HeLa). Phytother Res. 2021;35(11):6191–203. 34494317 10.1002/ptr.7255

[5] Mossmann D, Park S, Hall MN. mTOR signalling and cellular metabolism are mutual determinants in cancer. Nat Rev Cancer. 2018;18(12):744–57. 30425336 10.1038/s41568-018-0074-8

[6] Wang Y, Zhong J, Bai J, Tong R, An F, Jiao P, et al. The application of natural products in cancer therapy by targeting apoptosis pathways. Curr Drug Metab. 2018;19(9):739–49. 29749309 10.2174/1389200219666180511154722

[7] Tewari D, Patni P, Bishayee A, Sah AN, Bishayee A. Natural products targeting the PI3K-Akt-mTOR signaling pathway in cancer: a novel therapeutic strategy seminars in cancer biology. Amsterdam: Elsevier; 2019. 10.1016/j.semcancer.2019.12.008 31866476

[8] Rashed K, Ćirić A, Glamočlija J, Calhelha RC, Ferreira ICFR, Soković M. Antimicrobial and cytotoxic activities of Alnus rugosa L. aerial parts and identification of the bioactive components. Ind Crops Prod. 2014;59:189–96.

[9] Abo-Elghiet F, Mohamed SA, Yasin NAE, Temraz A, El-Tantawy WH, Ahmed SF. The effect of Alnus incana (L.) Moench extracts in ameliorating iron overload-induced hepatotoxicity in male albino rats. Sci Rep. 2023;13(1):7635. 37169909 10.1038/s41598-023-34480-6 PMC10175300

[10] Stević T, Šavikin K, Zdunić G, Stanojković T, Juranić Z, Janković T, et al. Antioxidant, cytotoxic, and antimicrobial activity of Alnus incana (L.) Ssp. incana Moench and A. Viridis (Chaix) DC Ssp. Viridis extracts. J Med Food. 2010;13(3):700–4. 20438323 10.1089/jmf.2009.0111

[11] Dahija S, Haverić S, Čakar J, Parić A. Antimicrobial and cytotoxic activity of Alnus glutinosa (L.) Gaertn., A. incana (L.) Moench, and A. viridis (Chaix) DC. extracts. J Health Sci. 2016;6(2):100–4.

[12] Lu Z, Liu H, Editorial. Molecular targets and therapeutic strategies in gynecological cancers. Front Oncol. 2022;12. 10.3389/fonc.2022.904032 PMC9047775 35494086

[13] Tian X, Srinivasan PR, Tajiknia V, Uruchurtu AFSS, Seyhan AA, Carneiro BA et al. Targeting apoptotic pathways for cancer therapy. J Clin Investig. 2024;134(14). 10.1172/JCI179570 PMC11245162 39007268

[14] Chaudhry G-e-S, Md Akim A, Sung YY, Sifzizul TMT. Cancer and apoptosis: the apoptotic activity of plant and marine natural products and their potential as targeted cancer therapeutics. Front Pharmacol. 2022;13:842376. 36034846 10.3389/fphar.2022.842376 PMC9399632

[15] van Meerloo J, Kaspers GJL, Cloos J. Cell sensitivity assays: the MTT assay. In: Cree IA, editor. Cancer cell culture: methods and protocols. Totowa, NJ: Humana; 2011. pp. 237–45. 10.1007/978-1-61779-080-5_20 21516412

[16] Da’i M, Meilinasary KA, Suhendi A, Haryanti S. Selectivity index of alpinia galanga extract and 1’-acetoxychavicol acetate on cancer cell lines. Indonesian J Cancer Chemoprevention. 2019;10(2):95–100.

[17] Abo-Elghiet F, Ibrahim MH, El Hassab MA, Bader A, Abdallah QMA, Temraz A. LC/MS analysis of Viscum cruciatum Sieber ex Boiss. Extract with anti-proliferative activity against MCF-7 cell line via G0/G1 cell cycle arrest: an in-silico and in-vitro study. J Ethnopharmacol. 2022;295:115439. 35667581 10.1016/j.jep.2022.115439

[18] Matthews HK, Bertoli C, de Bruin RAM. Cell cycle control in cancer. Nat Rev Mol Cell Biol. 2022;23(1):74–88. 34508254 10.1038/s41580-021-00404-3

[19] Sarkar S. Role of Paclitaxel and Vinblastine in Modern Cancer Therapy. Biswas HS, Progress in chemical and biological science [Internet]2023. pp. 15–25.

[20] Yu J, Zhong B, Xiao Q, Du L, Hou Y, Sun H-S, et al. Induction of programmed necrosis: A novel anti-cancer strategy for natural compounds. Pharmacol Ther. 2020;214:107593. 32492512 10.1016/j.pharmthera.2020.107593

[21] Cory S, Adams JM. The Bcl2 family: regulators of the cellular life-or-death switch. Nat Rev Cancer. 2002;2(9):647–56. 12209154 10.1038/nrc883

[22] Raisova M, Hossini AM, Eberle J, Riebeling C, Orfanos CE, Geilen CC, et al. The Bax/Bcl-2 ratio determines the susceptibility of human melanoma cells to CD95/Fas-mediated apoptosis. J Invest Dermatology. 2001;117(2):333–40. 10.1046/j.0022-202x.2001.01409.x 11511312

[23] Chresta CM, Masters JRW, Hickman JA. Hypersensitivity of human testicular tumors to etoposide-induced apoptosis is associated with functional p53 and a high Bax: Bcl-2 ratio. Cancer Res. 1996;56(8):1834–41. 8620501

[24] Gadducci A, Cosio S, Muraca S, Genazzani AR. Molecular mechanisms of apoptosis and chemosensitivity to platinum and Paclitaxel in ovarian cancer: biological data and clinical implications. Eur J Gynaec Oncol–IssN. 2002;392:2936. 12440809

[25] Dasari S, Bernard Tchounwou P. Cisplatin in cancer therapy: molecular mechanisms of action. Eur J Pharmacol. 2014;740:364–78. 25058905 10.1016/j.ejphar.2014.07.025 PMC4146684

[26] Coleman SC, Stewart ZA, Day TA, Netterville JL, Burkey BB, Pietenpol JA. Analysis of cell-cycle checkpoint pathways in head and neck cancer cell lines: implications for therapeutic strategies. Archives Otolaryngology–Head Neck Surg. 2002;128(2):167–76. 10.1001/archotol.128.2.167 11843726

[27] De Nys L, Barzegar-Fallah A, Lanckmans K, Steurbaut S, Beckwée D, de Haar-Holleman A, et al. Dose-Limiting toxicities of Paclitaxel in breast Cancer patients: studying interactions between pharmacokinetics, physical activity, and body Composition—A protocol for an observational cohort study. Cancers. 2024;17(1):50. 39796679 10.3390/cancers17010050 PMC11719000

[28] Sakthivel R, Malar DS, Devi KP. Phytol shows anti-angiogenic activity and induces apoptosis in A549 cells by depolarizing the mitochondrial membrane potential. Biomed Pharmacother. 2018;105:742–52. 29908495 10.1016/j.biopha.2018.06.035

[29] Song Y, Cho SK. Phytol induces apoptosis and ROS-mediated protective autophagy in human gastric adenocarcinoma AGS cells. Biochem Anal Biochem. 2015;4(4):1.

[30] Zafaryab M, Fakhri KU, Khan MA, Hajela K, Rizvi MMA. In vitro assessment of cytotoxic and apoptotic potential of palmitic acid for breast cancer treatment. Int J Life Sci Res. 2019;7(1):166–74.

[31] Fan H, Huang W, Guo Y, Ma X, Yang J. α-Linolenic acid suppresses proliferation and invasion in osteosarcoma cells via inhibiting fatty acid synthase. Molecules [Internet]. 2022; 27(9). 10.3390/molecules27092741 PMC9105512 35566090

