# Supplementary material for: Chemical profiling and anticancer activity of Alnus incana dichloromethane fraction on HeLa cells via cell cycle arrest and apoptosis
Source: BMC Complement Med Ther. 2025 May 26;25:189. doi: 10.1186/s12906-025-04920-z (PMC12105127; doi:10.1186/s12906-025-04920-z)
Supplement: Supplementary file 1 — Supplementary Material 1 [file 12906_2025_4920_MOESM1_ESM.pdf]

Researcher : Dr.Walaa Hesham email: Walaa.hesham91@gmail.com **mob.** 01023250543
Walaahesham35@yahoo.com 01551972143

Assay : Cell Cycle Analysis
Samples : 04 samples
cell line : ---
Ref. : ---
Date : 05-03-2024
Reader : BD FACSCalibur
Kit used : ab139418_Propidium Iodide Flow Cytometry Kit/BD
Solvent : DMSO
Assay samples : Cell culture

Lab Report

ser	Sample		DNA content			
	code	IC50 uM	%G0-G1	%S	%G2/M	Comment
1	Alnus/HeLa	---	58.22	29.38	12.4	cell growth arrest@ G1
2	cont.HeLa	---	47.42	35.76	16.82	---

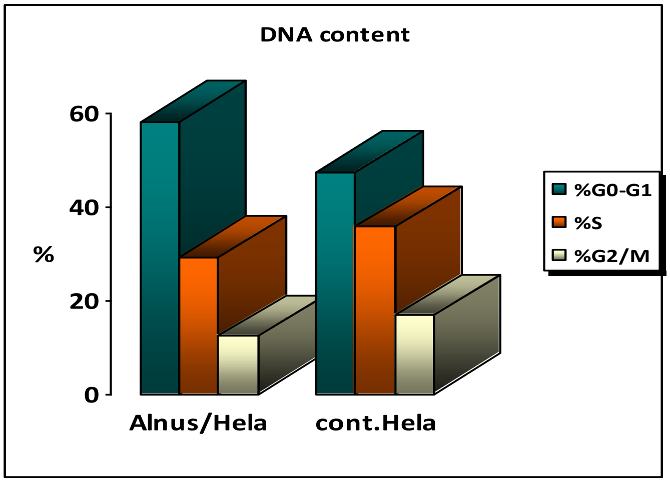

s	code	conc	Apoptosis			Necrosis
			Total	Early	Late	
1	Alnus/HeLa		21.55	14.32	4.39	2.84
2	Cont. HeLa		1.61	0.37	0.09	1.15

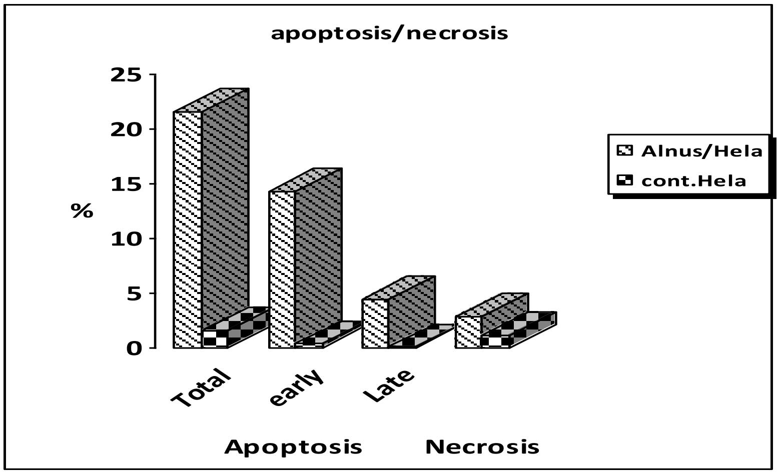

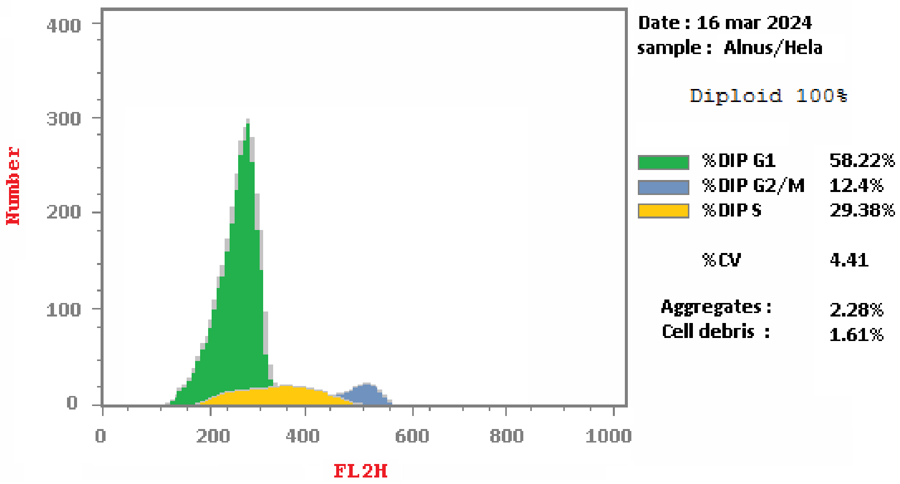
Date : 16 mar 2024
sample : Alnus/Hela
Diploid 100%
%DIP G1 58.22%
%DIP G2/M 12.4%
%DIP S 29.38%
%CV 4.41
Aggregates : 2.28%
Cell debris : 1.61%
Number
FL2H
0
100
200
300
400
600
800
1000

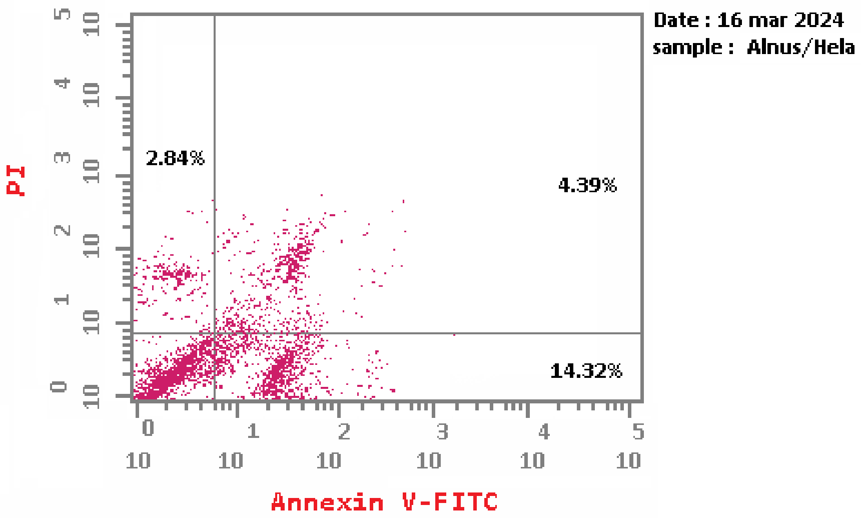
Date : 16 mar 2024
sample : Alnus/Hela
2.84%
4.39%
14.32%
PI
Annexin V-FITC

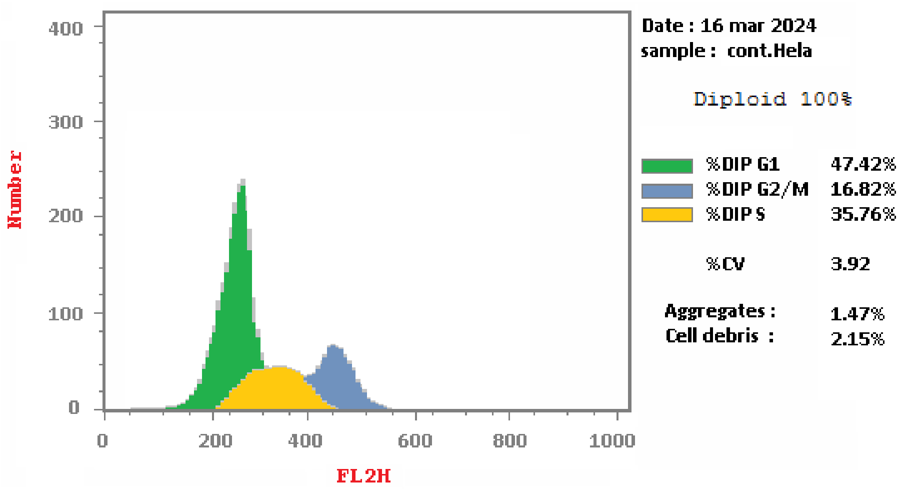
Date : 16 mar 2024
sample : cont.Hela
Diploid 100%
%DIP G1 47.42%
%DIP G2/M 16.82%
%DIP S 35.76%
%CV 3.92
Aggregates : 1.47%
Cell debris : 2.15%
Number
FL2H
0
100
200
300
400
0
200
400
600
800
1000

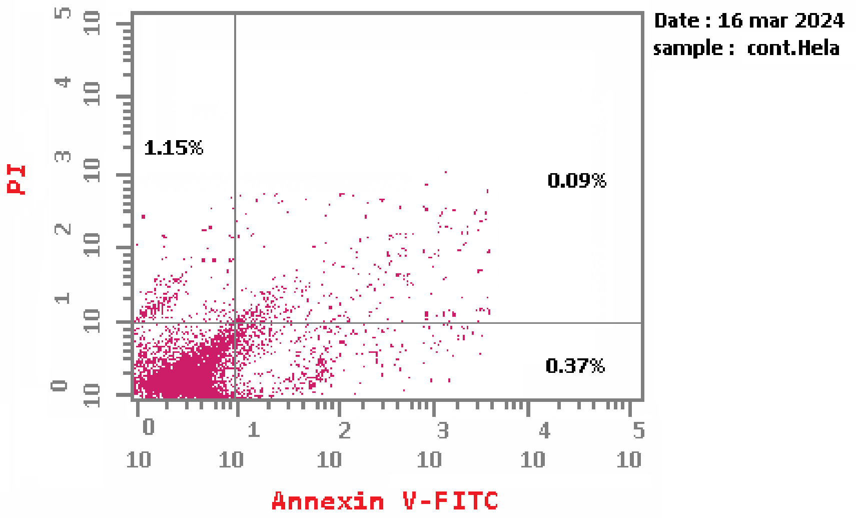
Date : 16 mar 2024
sample : cont.Hela
1.15%
0.09%
0.37%
PI
Annexin V-FITC
10^0
10^1
10^2
10^3
10^4
10^5
