# Supplementary material for: Chemical profiling and anticancer activity of Alnus incana dichloromethane fraction on HeLa cells via cell cycle arrest and apoptosis
Source: BMC Complement Med Ther. 2025 May 26;25:189. doi: 10.1186/s12906-025-04920-z (PMC12105127; doi:10.1186/s12906-025-04920-z)
Supplement: Supplementary file 2 — Supplementary Material 2 [file 12906_2025_4920_MOESM2_ESM.pdf]

**Researcher**	: Dr.Walaa Hesham	email: Walaa.hesham91@gmail.com	**mob.** 01023250543
**Assay**	: RT-PCR	Walaahesham35@yahoo.com	01551972143
**Samples**	: 04 Samples		
**Cell lines**	: ----		
**Ref.**	: ----		
**Date**	: 05-03-2024		
**Reader**	: Rotorgene RT- PCR system		
Kit used	: Qiagen RNA extraction/BioRad syber green PCR MMX		
**Solvent**	: DMSO		
**Assay samples**	: Cell Lysate		

Lab Report

**Ser**	**Sample**			**RT-PCR Fold Change**			
	**code**	**cells**	**conc**	**Bax**	**bcl2**	**p53**	
**1**	Alnus/HeLa	Hela	---	2.9606	0.6466	2.6025	
**2**	cont.HeLa	---	---	1	1	1	

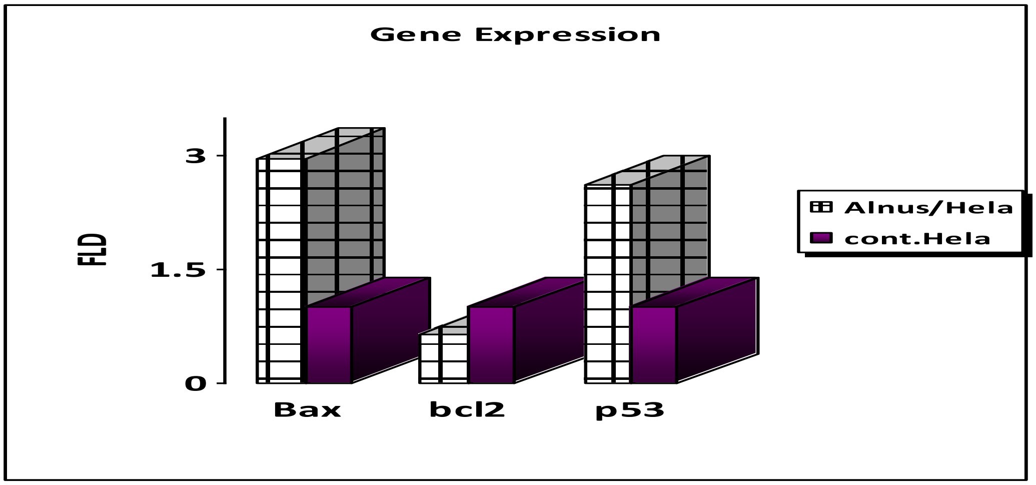

14 Hilly Street Mortlake NSW 2137 Australia
T + 61 2 9736 1320
F + 61 2 9736 1364
W www.corbettlifescience.com

Quantitation Report

Experiment Information

Run Name	Run 2024-03-16 (2)
Run Start	2024-03-16 05:13:22 PM
Run Finish	2024-03-16 07:41:12 PM
Operator	ERA
Notes	---
Run On Software Version	Rotor-Gene 1.7.87
Run Signature	The Run Signature is valid.
Gain Green	10.
Gain Yellow	9.33

Quantitation data

This report generated by Rotor-Gene 6000 Series Software 1.7 (Build 87)
Copyright ©2000-2006 Corbett Research, a Division of Corbett Life Science. All rights reserved.
ISO 9001:2000 (Reg. No. QEC21313)

[redacted]

primers

[redacted]

Bax : F 5’-TCAGGATGCGTCCACCAAGAAG -3’,
Bax : R 5'-TGTGTCCACGGCGGCAATCATC -3'.

[redacted]

bcl2 : F 5’-ATCGCCCTGTGGATGACTGAGT -3’,
bcl2 : R 5'-GCCAGGAGAAATCAAACAGAGGC -3'.

[redacted]

p53 : F 5’-CCTCAGCATCTTATCCGAGTGG -3’,
p53 : R 5'-TGGATGGTGGTACAGTCAGAGC-3'.

[redacted]

GAPDH : F 5’- GTCTCCTCTGACTTCAACAGCG-3’
GAPDH : R 5’- ACCACCCTGTTGCTGTAGCCAA-3’

Sample	Gene Expression		
	Control cells	Test cells	FLD

Ser	code	Conc	GAPDH	Bax	ΔCTC	GAPDH	Bax	ΔCTE	ΔΔ CT	2^ ΔΔCT
			HC	TC	TC-HC	HE	TE	TE-HE	ΔCTE-ΔCTC	E=1.866
**1**	**Alnus/ HeLa**		**22.41**	**37.93**	15.52	**22.29**	**36.07**	13.78	**-1.74**	2.9606
**2**	**cont. HeLa**		**22.41**	**37.93**	15.52	**22.41**	**37.93**	15.52	**0**	1

Ser	code	Conc	GAPDH	Bcl2	ΔCTC	GAPDH	Bcl2	ΔCTE	ΔΔ CT	2^ ΔΔCT
			HC	TC	TC-HC	HE	TE	TE-HE	ΔCTE-ΔCTC	E=1.866
**1**	**Alnus/HeLa**		**22.41**	**27.96**	5.55	**22.29**	**28.55**	6.26	**0.71**	0.6466
**2**	**cont. HeLa**		**22.41**	**27.96**	5.55	**22.41**	**27.96**	5.55	**0**	1

Ser	code	Conc	GAPDH	p53	ΔCTC	GAPDH	p53	ΔCTE	ΔΔ CT	2^ ΔΔCT
			HC	TC	TC-HC	HE	TE	TE-HE	ΔCTE-ΔCTC	E=1.839
**1**	**Alnus/ HeLa**		**22.41**	**36.71**	14.3	**22.29**	**35.02**	12.73	**-1.57**	2.6025
**2**	**cont. HeLa**		**22.41**	**36.71**	14.3	**22.41**	**36.71**	14.3	**0**	1
